# Hedonic Analysis of Dried Pasta Prices Using E-Commerce Data—An Explorative Study

**DOI:** 10.3390/foods13060903

**Published:** 2024-03-16

**Authors:** Francesco Bimbo, Emilio De Meo, Domenico Carlucci

**Affiliations:** 1 Department of Agricultural, Food, Natural Resources and Engineering Sciences (DAFNE), University of Foggia, Via Napoli, 25, 71122 Foggia, Italy; 2 Department of Soil, Plant and Food Science (Di.S.S.P.A.), University of Bari Aldo Moro, Via G. Amendola, 165/a, 70126 Bari, Italy; emilio.demeo@uniba.it (E.D.M.); domenico.carlucci@uniba.it (D.C.)

**Keywords:** durum wheat dried pasta, hedonic price model, implicit prices

## Abstract

Italy is the world leader in the production of pasta and the Italian market is characterized by strong price competition among large industrial producers. Thus, recently, many small and medium firms have started to differentiate their products as a way to achieve higher margins and escape from price competition. Using data on the prices and characteristics of dried pasta sold online in the Italian market and a hedonic price model, we estimated the implicit prices associated with several attributes that are currently available for dried pasta. We find that the “artisanal” statement on pasta labeling is associated with the highest price premium. Also, results show that protected geographical indication, Halal and Kosher certifications, and the use of ancient wheat varieties are valuable features of dried pasta sold in the Italian market. Instead, a positive, albeit limited in magnitude, price premium is associated with dried pasta made using 100% Italian durum wheat semolina, the organic method, enriched with additional ingredients. Findings suggest that producers can differentiate their products by mostly emphasizing their small-scale production methods, the territorial connotation, and the cultural and environmental sustainability of production. Otherwise, certifying dried pasta as Halal or Kosher can represent a complementary or alternative strategy to differentiate the product and achieve a higher price.

## 1. Introduction

Dried pasta made from durum wheat is one of the most consumed wheat-based foods worldwide due to its sensorial properties, nutritional value, affordability, and extended shelf life at room temperature. Additionally, dried pasta is very easy to prepare [1]. According to the International Pasta Organization, global pasta production has doubled in the last 20 years; it is valued at approximately EUR 20 billion, and its market is predicted to expand by 2.3% until 2025 [2].

Italy is the world leader in the production of pasta, contributing 25% of global production in volume, followed by the U.S. and Turkey. Also, Italy is the country with the highest per capita pasta consumption (23.5 kg/year, followed by Tunisia with 17 kg/year). Historically, the Italian industry of dried pasta is highly concentrated and dominated by a few large companies that mainly compete on price. This market structure makes it difficult for small local producers to enter such a market and lowers the profit margins of large companies [3,4,5]. Thus, over the past few years, a rising share of Italian dried pasta producers have adopted a product differentiation strategy that, by satisfying the changing preferences of modern consumers, allows them to obtain higher market prices and escape from price competition that characterizes this market [1]. Furthermore, these manufacturers often opt for e-commerce platforms to sell their innovative products, overcoming the hurdle of large retailers that face challenges in expanding the variety of products offered in their physical stores [4,6].

However, over the past decade, the marketing literature has marginally explored the consumer behavior for dried pasta features. Studies have analyzed consumers’ attitudes, acceptance, and preferences for several of pasta’s characteristics across various geographic locations, such as Italy, Poland, Australia, etc. A wide array of research methods, encompassing both qualitative (e.g., focus groups) and quantitative (e.g., means–end analysis and choice-based analysis) techniques, were employed [7,8,9,10,11,12,13,14,15,16,17,18,19,20,21,22]. These studies found that consumers primarily base their pasta choices on a few attributes, like wheat origin, brand, and price [8,13,19,20,22]. In detail, Italian consumers show a strong preference for pasta made from Italian durum wheat. This is associated with a higher perceived quality, especially if it has a certified origin, like the protected geographical indication (PGI) [19,20,22]. Also, Italian consumers often equate Italian pasta brands with “Italian wheat”, but this is not always the case [22]. Furthermore, consumers highly value organic pasta, as well as pasta produced using whole wheat semolina and ancient durum wheat varieties, such as “Senatore Cappelli”, due to a mix of environmental, health, and cultural reasons [8,9,11,12,18,19,20,21]. Studies also found a rising consumer interest in pasta labeled “artisanal”, which is produced by small firms, in limited quantity using some handmade and traditional practices, such as slow drying or hand drawing [7,11,19,20]. Other studies investigated consumers’ acceptance of dried pasta with additional ingredients, such as eggs, tomatoes, spinach, etc. [10,15]. Lastly, and more recently, scholars recorded a rising demand for pasta with Halal and Kosher certifications, offering assurance that the product was manufactured in compliance with the precepts of Islamic and Jewish law, respectively, as a result of the growing multiculturalism in modern societies [16].

However, it is worth noting that the studies conducted on consumer preferences primarily relied on self-reported data. These data were obtained by asking consumers to express their attitudes, perceptions, and purchase intentions toward products that had specific characteristics. Due to the subjective nature of product evaluations and the use of multiple metrics across these studies, there is high variability in the results, making it difficult to compare them. Additionally, the findings may only apply to the specific group of consumers surveyed, and there is no guarantee that what a person expresses in a focus group, interview, or choice experiment aligns with their real-life behavior. Therefore, in our analysis, we used market prices as an objective metric to gauge the performance of the different quality attributes of dried pasta.

To achieve this, we employed an analytical approach based on the hedonic price model, wherein product attributes are assessed by considering their impacts on the product market price, the so-called implicit market prices. The hedonic price model is widely used by food economists to estimate the market value, or implicit price, of specific attributes of food products. This approach benefits producers that can use implicit prices to develop marketing strategies and select the optimal mix of attributes aware of their costs of production. Also, it helps to isolate premiums for “credence attributes”, like organic certification, which consumers cannot verify even after repeated purchases. Policymakers can use hedonic price analysis to understand producer incentives related to credence attributes and potential issues like false claims or free riding on the name of a geographical area known for providing high-quality food products (e.g., “Parma” ham, “Chianti” wine). Compared to other methods, like choice-based analysis, surveys, and focus groups, the hedonic price analysis offers the advantages of analyzing real market data with results that reflect the actual consumer choices by mitigating hypothetical bias and generating robust and comparable findings across studies. Therefore, hedonic price analysis is a valuable tool for understanding aggregate consumer preferences, informing producers about profitable differentiation strategies, and supporting policy decisions in the food market [23].

To employ the hedonic price model, the current study used data on the prices and characteristics of dried pasta sold in the Italian market and retrieved from Amazon.it, the largest e-commerce marketplace offering a wide assortment of products, including food staples and pasta. To the best of our knowledge, there has been no prior investigation aimed at estimating the implicit prices associated with the attributes of dried pasta using real market data. Indeed, in the past decade, manufacturers have pursued product differentiation strategies by introducing dried pasta with new features on the market. However, despite the availability of new features associated with dried pasta, a comprehensive analysis of their market performance when analyzed together is still unknown. Therefore, measuring the implicit market prices of the various dried pasta features could be informative for companies undertaking product differentiation strategies aimed at increasing their revenues.

The remainder of this paper is organized as follows. The next subsection describes the Italian market of dried pasta; Section 2 describes the theoretical model, data collected, and econometric estimation; Section 3 discusses the empirical findings; finally, Section 4 concludes the manuscript by providing some marketing recommendations for Italian producers, as well as future research avenues.

### Italian Supply of and Demand for Pasta

The Italian pasta market records 4624 pasta-producing companies in 2022, of which approximately 80% are small–medium-sized firms. Overall, pasta-producing companies employed 27,510 workers and generated an annual revenue of approximately EUR 7.5 million in 2022 by selling 3.27 million tons of pasta. According to the market data available and reported in Table 1, there has been a 10.5% decrease in the overall number of pasta firms operating in the Italian market between 2012 and 2022, even though there has been a relevant increase in production (+40%). This is due to several acquisitions and mergers of companies that occurred in this market in an attempt to benefit from economies of scale, higher efficiency, lower production costs, and higher margins [5,24,25,26].

The Italian market of pasta is dominated by 10 industrial companies listed in Table 2 that generate a turnover of approximately EUR 6 million, accounting for about 80% of the total industry turnover in 2022.

Italian pasta producers largely source durum wheat from other countries, as durum wheat produced nationally is not enough to meet the Italian pasta industry’s needs. In 2022, Italy imported over 2.2 million tons of durum wheat, mainly from Canada, the United States, and Australia. Instead, pasta produced in Italy is largely exported abroad. In 2022, over 1.89 million tons of pasta produced in Italy was exported to almost 200 countries worldwide. Germany is the first export market followed by France and the United States. Also, pasta exports to Spain, Belgium, and Eastern countries are growing [3,5,27].

On the demand side, Italians are among the highest consumers of dried pasta per person in the world. They consume an average of 23.5 kg annually, with about half of the country’s population, which is approximately 60 million people, declaring to eat pasta every day. In the past decade, the demand for various types of pasta in Italy has been on the rise, including whole flour pasta, organic pasta, as well as pasta made with alternative flours, and legumes, like pea flour, lentils, and chickpeas [3]. Consumers are also placing a strong emphasis on the country from which the raw material is produced, such as the case of the 100% made-in-Italy pasta, supporting the growth in market shares of Italian producers that use Italian durum wheat semolina [3,5,22]. Thus, the demand is largely oriented to products with additional features (e.g., organic, “100% Italian”) compared to the conventional pasta historically consumed in the Italian market. Also, the purchasing behaviors are changing, and there are a growing share of consumers who shop for pasta online where artisanal pasta options produced by small local producers are available. Indeed, e-commerce in Italy has been on the rise, with more consumers turning to online shopping for various products, including food items like pasta. The growth of online shopping for food items in Italy can be attributed to factors like convenience, greater product variety, and the COVID-19 pandemic. Several e-commerce platforms and grocery delivery services in the country offer pasta and other food items for purchase online [5].

## 2. Materials and Methods

### 2.1. Data Collection and Description

Data on the prices and characteristics of dried pasta sold online in the Italian market was collected via direct observation of the website Amazon.it, which is the largest e-commerce marketplace in Italy, offering a wide assortment of products, including food staples. Data search and collection were carried out in December 2022. Starting from the homepage of Amazon.it, we used “dried pasta” as a keyword to search for products available for sale. Amazon’s engine then returned 260 results organized into 6 web pages that were simultaneously saved. Each result was related to a specific dried pasta product available for sale, identified by a picture and a brief description. By clicking on each result, Amazon’s search engine provided detailed information on the selected product, as well as a readable copy of its label. So, all product details were carefully extracted and recorded in a database. Specifically, for each dried pasta product, we collected information on its price, the lot size (minimum quantity order), and whether the product was sold under the Prime scheme (fast delivery within one day and shipping fees included in the price), free shipping fees scheme (no fast delivery but shipping fees included in the price), or ordinary scheme (no fast delivery and shipping fees not included in the price). Also, we gathered information on the package size, brand, whether the product was labeled “artisanal”, whether the product was bronze drawn, whether the product was made with whole wheat semolina or had other ingredients added (e.g., tomatoes, spinaches, beans, peas, etc.), whether the product was made from ancient wheat varieties, such as “Senatore Cappelli”, whether the product was made from 100% Italian durum wheat, and whether the product had organic certification, geographical indications, and/or Kosher/Halal certification. The product features collected and their summary statistics are listed in Table 3. The final database encompasses information on 260 dried pasta products sold under 63 brands.

### 2.2. Empirical Model and Statistical Analysis

In this paper, we utilized the standard hedonic price model developed by Rosen [28]. Rosen’s framework implies that every consumer in the market purchases a product that offers the best combination of the product’s features and maximizes his/her utility, conditionally on his/her budget constraint. Likewise, each producer sets the price of their products based on the attributes included in the product itself. In a market where each product represents a unique bundle of attributes, at equilibrium, consumer willingness to pay for the product offered and producer willingness to accept the product sold will match. This generates a hedonic price function, where the price of product *j* in market *m* and $P_{jm}$ can be described by the following function:

(1) $$P_{jm}=f(X_{jm})$$

where *X* is a vector of product characteristics and *f*(.) is an unspecified functional form. Equation (1) indicates that product price *j* in market *m* embeds the marginal monetary values of *j*’s attributes [28], and the marginal monetary value of *j* that can be obtained by partially differentiating (1) with respect to each attribute. In the current study, *X* encompasses dried pasta features listed in Table 3 along with brand-fixed effects.

According to the previous literature parameters in Equation (1) were estimated using a single equation approach via Ordinary Least Squares (OLS) estimator. The dependent variable, the pasta price, was transformed into its logarithm, and robust standard errors were used in the estimation process. Also, the implicit price of each dichotomic variable was calculated using Kennedy et al.’s [29] adjustment. Agricultural economists have extensively used the hedonic price model to estimate the market value of several food products’ attributes primarily associated with different products, such as apple size and grades [30], tuna qualities [31], wheat chemical composition [32], organic product premiums and geographical indications for meat [33], and extra virgin olive oil [34]. Estimates are reported in Table 4. The variables included in the model are in the first column, the estimated parameters associated with each variable with standard errors in parentheses are in the second column, and the implicit prices of each product’s characteristics in percentage are reported in the third and last column.

## 3. Results and Discussion

The estimated parameters in Equation (1), using the logarithmic transformation of the price as the dependent variable, are reported in the first column in Table 4, with standard errors in parenthesis. Marginal prices of each attribute (in percentage terms) are calculated using Kennedy’s (1981) adjustment and reported in the last column in Table 4. The baseline product is dried pasta obtained from imported durum wheat, sold in packages of 500 gr, without any certifications, artisanal labeling, or other ingredients added. The model shows a high adjusted R^2^ and a value of the Variance Inflation Factor (VIF) below 10, suggesting low multicollinearity and that regression coefficients are not significantly affected by correlations between independent variables, ensuring reliable regression coefficient estimates. Furthermore, based on Ramsey’s RESET [35] statistics for omitted variable bias, the model does not suffer from misspecification.

First, estimated results for e-commerce seller scheme variables show that the Prime-eligible pasta product has a premium price of +66.89% compared to the baseline product charged with shipping fees or sold under the free shipping fees scheme. Prime products are associated with higher prices, as they attract customers willing to benefit from fast shipping, which prompts some buyers to pay a premium for Prime-eligible items compared to non-Prime items, which have longer shipping times. This perceived higher value of Prime products contributes to their higher price [36,37]. Products sold under the free shipping fees scheme do not record higher prices compared to the product baselines. It is important to say that Amazon’s pricing is dynamic, and it can change frequently based on market conditions, seller decisions, and other factors. As a result, the relationship between shipping fees and product prices can vary across different sellers on the platform. The *Lot_size* variable is also significant, with a coefficient equal to −0.26. Taking into account the logarithmic form of the equation, this coefficient should be interpreted in terms of elasticity. Specifically, the negative (but less than one) coefficient means that a discount on the unit price is given when a larger amount of pasta is purchased in a single order.

Second, regarding the estimated results for the product’s features, it is important to notice that the marginal prices associated with the packaging variables show statistically significant coefficients. Premium prices are associated with pasta products sold in packages smaller than 500 gr (“*Package_size_less500gr*”) benefiting from a premium price of +35.23%, compared with the product baseline. Also, interesting results are found for pasta labeled “artisanal”, which is associated with a price premium of +125.74%, equal to a premium price of EUR 9.9/kg compared to the product baseline. The high price of artisanal pasta is due to several concurrent reasons. First, individuals overall perceive artisanal pasta as a higher quality pasta compared to the conventional one produced by large-scale industrial factories; second, small producers prioritize traditional production methods, relying on meticulous craftsmanship and manual work, which may attract consumers willing to pay for traditional products [38,39]; third, local producers have limited production capacity, which results in a restricted supply of artisanal pasta products, which drives up prices due to the combination of limited supply and high demand; fourth, and last, the higher price of artisanal pasta is likely due to consumers’ willingness to support local businesses and communities by purchasing artisanal pasta products [38,39,40].

Also, a notable finding reported in Table 2 is that the “*Italian*” origin label has a positive and significant effect on the price of pasta and amounts to a price premium of +17.76% relative to the baseline product, which is equivalent to a premium of EUR +1.399/Kg. This result is consistent with other studies that found a willingness of Italian consumers to pay more for domestic food products, such as extra virgin olive oil (EVOO) [41], wine [42], and milk [43,44]. In detail, Italians likely prefer 100% Italian pasta due to the higher food safety standards enforced in Italy and the EU. This aligns with Italy’s aversion to the risk of glyphosate and mycotoxins in non-Italian grains, which are common contaminants in pasta from outside the EU. The stringent EU food safety regulatory schemes also foster trust toward products obtained from domestic ingredients and make the 100% Italian pasta a preferred choice for Italian consumers [19]. Instead, bronze-drawn pasta “*Bronze*”, produced using bronze molds, did not receive a price premium in our analysis despite its rough texture, which may appeal to consumers seeking superior culinary experiences [7]. It is likely that online shoppers may not immediately recognize the qualities associated with bronze-drawn pasta due to the presence of multiple pasta features on the label, leading to an information overload. Also, many online shoppers may not be familiar with the bronze-drawn pasta production method and its impact on pasta quality. As a result, online shoppers may not understand the benefits of this method and may not be willing to pay a premium.

Furthermore, dried pasta obtained with whole wheat semolina and/or enriched with additional ingredients “*Whole_wheat_and_with_added_ingredients*” receives a price premium of +9.82% relative to the baseline product, which is equivalent to a price premium of EUR +0.77/kg. Such types of pasta gained popularity over the last decade due to two main factors. First, such pasta options are prized for their superior nutritional value by health-conscious consumers. Indeed, whole wheat pasta provides higher fiber content and essential vitamins and minerals, while pasta with ingredients, like lentils and peas, has higher protein content and higher nutritional contents [11,20]. Second, the latter pasta varieties match specific dietary preferences, particularly vegetarianism and veganism. With plant-based diets on the rise, pasta added with legumes and vegetables becomes an attractive protein source for individuals attempting to limit or avoid animal products [21,45].

Pasta from ancient wheat varieties “*Ancient_variety”,* like Senatore Cappelli, is associated with a higher market price of about +31.45%, relative to the baseline product, equivalent to a price premium of EUR +2.47/kg. Related to this result, the literature offers several compelling reasons. Ancient grains provide peculiar nutritional features (e.g., high fiber, lower gluten) and a unique taste, attracting the interest of health-oriented consumers and those looking for culinary experiences [46]. Also, an ancient grain with its rich heritage ties into cultural traditions, appealing to those valuing culinary legacy [47]. Lastly, the environmental sustainability of these grains, needing less water and fewer pesticides, aligns with the eco-friendly food choices of sustainable consumers [37,47]. All these factors collectively justify the premium price recorded and the rising consumption of pasta from ancient grain varieties.

Interestingly, the “*Organic*” attribute achieves a positive and significant impact on the price of +10.42% over the baseline product price, equivalent to a price premium of EUR 0.821/kg. The premium price associated with this attribute is likely to be the result of existing shares in the market of consumers willing to pay for an “environmentally friendly” product, as reported in some studies [19,48]. Also, products labeled organic are often perceived as healthier than regular ones, and, indeed, consumers’ primary reason for buying organic foods is their belief that these products support human health [49,50,51].

The geographical indication “*GIs*” records a positive and significant effect on the pasta price of EUR +3.2/kg, or +40.69%, relative to the baseline product’s price. The result is consistent with several studies that also found that consumers, including those in the Italian market, prefer GI products over regular ones and are willing to pay higher prices for such products [52], including pasta [7,19].

Remarkably, pasta with Halal or Kosher certification “*Halal_Koscher_certification*” receives higher market prices of +36.41%, or EUR +2.87/kg. This is likely due to the growing demand among Islamic and Jewish consumers for specialized Italian foods, including pasta, as suggested by De Boni and Forleo [16]. The rising pasta demand among such communities significantly influences the entire sector of Italian dried pasta. Indeed, the increasing availability of Halal- and Kosher-certified pasta is perceived as a driving force for the market expansion of high-quality Italian dried pasta [16]. However, it is worth mentioning that the higher price of pasta with organic, geographical indications, or Halal and Kosher certifications may reflect the higher cost of those pasta versions since farmers/producers seeking to sell their products with such labels have to meet costly production standards and certifications fees.

## 4. Conclusions

This study points out that dried pasta sold in the Italian market and through e-commerce channels is surprisingly highly differentiated, and its selling price is strongly affected by some characteristics that are not commonly found in dried pasta sold in conventional physical stores. Dried pasta differentiation is based on several factors, such as the use of durum wheat semolina with a domestic origin, the use of semolina obtained from ancient durum wheat varieties, like “Senatore Cappelli”, and the use of whole wheat semolina and/or additional ingredients, like eggs, tomatoes, spinach, and more. Furthermore, some products are sold with the “artisanal” label, suggesting to consumers that the dried pasta is made using traditional and manual methods, which may involve the use of bronze molds for shaping the pasta, and some steps in the production process are performed by hand. Also, dried pasta is differentiated using certifications, including organic, PGI, and Kosher/Halal.

Product differentiation has a significant impact on the selling price of dried pasta. The price range is wide, with the minimum being EUR 1.49/kg and the maximum being EUR 36.00/kg. Our econometric analysis, which was based on the estimation of a hedonic price model, showed that, except for bronze-drawing dried pasta, all the attributes mentioned above add a premium to the product. Specifically, the use of whole wheat semolina and/or additional ingredients adds a premium of 9%, organic certification adds 10%, the use of durum wheat semolina with Italian origin adds 17%, semolina from ancient durum wheat varieties adds 31%, Kosher/Halal certification adds 36%, PGI certification adds 40%, and finally, dried pasta labeled “artisanal” adds a premium of 125% compared with the average price. These findings can be useful for dried pasta manufacturers aiming to adopt product differentiation strategies. In detail, manufacturers may compare the premium prices associated with each quality feature of dried pasta with the costs needed to incorporate it in the final product and then select an optimal mix of features that is both feasible and profitable.

Our findings suggest that online sales can be a significant benefit for small-scale artisanal pasta producers, as the “artisan” attributes of dried pasta command the highest price premium in the e-commerce market. Also, online platforms allow small-scale producers to sell directly to customers, bypassing traditional distribution channels and potentially earning more profit by reaching specific groups interested in artisanal products. Marketing pasta through online stores is also cheaper overall than selling it in physical stores, making its sales more accessible for smaller producers with limited resources. However, online sales come with challenges, such as building trust online. Producers may be willing to invest in trust-building activities (e.g., brand storytelling, loyalty programs and rewards, customer reviews and testimonials, open communication) to facilitate their sales. Despite these challenges, selling pasta online is a great opportunity for small-scale producers to expand their market and increase their profits.

The findings of this study can also provide valuable insights for policymakers. This study, in fact, reveals that the highest price premium is associated with dried pasta that is labeled “artisanal” (+125%), which attracts many consumers due to its perceived high quality and for which they are willing to pay more than double the price paid for conventional pasta.

However, to the best of our knowledge, there is a lack of legal definition of the “artisanal” term when it appears on the label of dried pasta. Few producers associate “artisanal” statements on the label with specific practices, such as slow drying or hand drawing, but most of them do not provide any further information. This is because there is no mandatory regulation that defines the standards for using the term “artisanal” on pasta labels. The void of a legal definition of the term “artisanal” may lead some companies to label their products “artisanal” without adhering to any standard or incurring additional costs, while still benefiting from the higher revenue generated by the premium price associated with such a feature. This lack of definition may create unfair competition where producers who genuinely follow artisanal methods (like slow drying or hand-drawing) compete with those who simply use the label for marketing purposes. Therefore, it would be highly desirable for this market to establish a clear definition of what “artisanal” pasta means, similar to the regulation of craft beer, to ensure fair competition among producers as well as guarantee consumers the right to receive non-misleading information from the label. Related to this point, implementing a legal definition for “artisanal” pasta might involve collaboration between industry groups, regulatory bodies, and consumer protection agencies. Producers’ associations and organizations can work together to establish clear criteria for artisanal production. Government agencies involved in food labeling can review and potentially adopt the industry-developed standards, while consumer protection groups can ensure the defined standards are clear and effectively communicated to the consumer. By working together, stakeholders in the dried pasta market can establish a system that benefits everyone: producers operating with integrity, consumers making informed choices, and a market characterized by fair competition and trust.

In spite of our results’ usefulness in providing guidance to pasta producers in deciding whether to invest in a differentiation strategy, our analysis has some limitations that will be addressed in future follow-up studies. Firstly, our results refer to the Italian e-commerce market, for which we use data retrieved from Amazon. Thus, future research will include other e-commerce portals, such as those of physical food retailers, to deepen the knowledge of market preferences from durum pasta attributes. Secondly, our results measure the average price contributions of durum wheat pasta features, neglecting potential non-linear relationships and synergic effects of product attributes on price. Thus, future research will utilize detailed household purchase data, along with flexible models and estimation techniques, like quantile regression, to address these limitations. Thirdly, a comparison of premium prices and relative costs associated with each attribute would offer a better picture of the actual markup achieved by adopting alternative differentiation strategies. Fourthly, and lastly, our results do not provide insights into the role played by consumers’ heterogeneity in the process of price formation, as obtained using aggregate data. Therefore, more in-depth analysis may be conducted to investigate consumer reasons and attitudes for purchasing durum wheat pasta and its related attributes.

## Figures and Tables

**Table 1:** Italian pasta industry data.

	2012	2013	2014	2015	2016	2017	2018	2019	2020	2021	2022	% Var. 2012–22
Pasta firms (n.)	5164	5108	5018	4960	4926	4889	4852	4790	4732	4679	4624	−10.45
Large	750	761	775	798	820	838	863	909	947	970	971	29.39
Small–Medium	4414	4348	4243	4163	4106	4051	3989	3881	3786	3708	3653	−17.23
Direct employees (n.)	25,924	25,424	24,793	25,237	26,102	26,024	26,496	27,182	27,130	27,170	27,510	6.12
Production (mln. ton.)	2.33	2.12	1.94	1.96	2	1.93	2.28	2.45	2.51	3.68	3.27	40.25

Source: our elaboration using data retrieved by Elenco aziende con fatturato per il codice ATECO 10.73 and the Chamber of Commerce of Marche region—open data [24,25,26].

**Table 2:** Leader companies in the Italian pasta market.

Rank	Company Name	Region	Revenues (Millions of Euros)
1	Barilla G. e R. Fratelli Società Per Azioni	Emilia Romagna	3230.00
2	F.lli De Cecco Di Filippo Fara San Martino Spa	Abruzzo	620.36
3	Pastificio Rana Spa	Veneto	595.07
4	F. Divella Spa	Apulia	356.19
5	Pastificio Lucio Garofalo Spa	Campania	259.77
6	La Molisana Spa	Molise	253.82
7	Pasta Zara Spa	Veneto	218.63
8	De Matteis Agroalimentare Spa	Campania	216.13
9	Rummo Spa Società Per Azioni	Campania	152.93
10	Bertagni 1882 Spa	Trentino Alto Adige	141.76

Source: Registro aziende [26].

**Table 3:** Summary statistics and variables description (Obs. = 260).

Variables	Variables Description	Mean ^a^
*Price*	Pasta price (EUR/kg)	7.877 (min 1.49–max 36.00; sd. 5.39)
*Prime*	1 = product sold under *Prime* scheme	0.210
*Free_shipping_fees*	1 = product sold under free shipping fees scheme	0.544
*Package_size_less500gr*	1 = pasta in packages less than 500 gr.	0.114
*Lot_size*	1 = quantity of pasta ordered for delivery (kg)	7.20 (min 5.52–max 9.39; sd. 1.00)
*Artisanal*	1 = pasta labeled artisanal	0.616
*100%_Italian*	1 = pasta made with 100% Italian wheat	0.796
*Bronze_drawn*	1 = pasta made with a bronze drawn system	0.758
*Whole_wheat_and_* *with_added_ingredients*	1 = pasta made with whole wheat flour or added with other ingredients	0.226
*Ancient_variety*	1 = pasta made with ancient wheat varieties	0.134
*Organic*	1 = pasta with organic certification	0.130
*Geographical_indications*	1 = pasta with PGI certification	0.184
*Halal_Kosher_certifications*	1 = pasta with Kosher or Halal certification	0.073

^a^ For all binary variables, the mean represents the percentage of observations showing a value of 1 and the standard deviation is omitted.

**Table 4:** Estimated parameters and percentage of premium price.

Variable	*β*		Percentage Premium Price
*Prime*	0.514	***	66.89
	(0.0931)		
*Free_shipping_fees*	0.485		
	(0.4114)		
*Package_size_less500gr*	0.311	**	36.23
	(0.1235)		
*Lot size*	−0.2620	**	
	(0.1173)		
*Artisanal*	0.8193	***	125.74
	(0.1116)		
*100%_Italian*	0.164	***	17.76
	(0.0572)		
*Bronze_drawn*	0.127		
	(0.097)		
*Whole_wheat_and_with_added_ingredients*	0.094	**	9.82
	(0.0390)		
*Ancient_variety*	0.275	**	31.45
	(0.1144)		
*Organic*	0.099	*	10.42
	(0.0503)		
*Geographical_indications*	0.342	***	40.69
	(0.0503)		
*Halal_Kosher_certification*	0.315	***	36.41
	(0.1771)		
*Constant*	0.995	***	
	(0.1892)		
R-square	0.905		
Variance Inflation Factor (VIF)	6.44		
*Specification test*			
Ramsey’s RESET F(3, 191)	1.42		
*p*-value	0.237		

Note: *, ** and *** are 10. 5 and 1% significance levels.

## Data Availability

The original contributions presented in the study are included in the article, further inquiries can be directed to the corresponding author.
